# Editorial: Individual Differences in Cognition and Affects in the Era of Pandemic and Machine Learning

**DOI:** 10.3389/fpsyg.2022.848086

**Published:** 2022-02-25

**Authors:** Andrea Vranic, Yang Jiang, Xiaopeng Zhao

**Affiliations:** ^1^Department of Psychology, Faculty of Humanities and Social Sciences, University of Zagreb, Zagreb, Croatia; ^2^Department of Behavioral Science, College of Medicine, University of Kentucky, Lexington, KY, United States; ^3^The University of Tennessee, Knoxville, Knoxville, TN, United States

**Keywords:** COVID-19, individual differences, anxiety, adherence to recommendations, negative affect, socio- cognitive factors

At the launch of this issue, then newly discovered COVID-19 seemed to be faltering, and the collection of papers gathered in this publication was bound to be both a reminder and a relatively final display of domains and aspects of human functioning that were somehow affected by the corona crisis. Meanwhile, the health situation in the world has shown us the full breadth of the impact that the coronavirus has left or still leaves in almost all fields of human behavior. Overall, bibliographic mapping shows that most of the papers, framed within the scientific study of coronavirus and its societal impact, were in the field of medicine and psychology (Gul et al., 2020). Mirroring the solidarity and unifying efforts to overcome the crisis, collaborative research and multiple authorships prevail. This exact pattern can be seen in the collection of 16 collaborative papers gathered within this Research Topic.

The Topic reported some surprising findings as well. For instance, counter to most people's initial expectation, young people suffered significantly more psychological stress than older people during the pandemic. Older adults who are more vulnerable to COVID-19 showed strong resilience around the world. Notably, the research on predictive model of stress and anxiety for depression in undergraduate students by Rodríguez-Hidalgo et al. has been read more than 113,000 times up to the point of the publication of this Editorial. Our readers are from all continents and in humanities, social, science, and health domains. Here, we summarize what we have learned from this Research Topic, in terms of differential psychological and emotional responses of young and older adults, as well as from emerging techniques aimed at coping with nowadays everyday pandemic.

As in the case of other health crises in the human history, our research collection has placed an emphasis on the effects of this pandemic on our emotional and cognitive behaviors, as well as attempts to prevent or mitigate its destructive effects. Indeed, most of the collected papers revealed the affective side of living with the pandemic. These studies cover topics of anxiety (Camacho et al.; Rodriguez-Hidalgo et al.), mental health (Osimo et al.; Mehulić and Kamenov), and positive (e.g., gratitude, purpose in life) and negative affects (Bernabe-Valero, Melero-Fuentes et al.; Hou et al.) during the COVID-19 outbreak. Taking a more differential approach, papers dealing with the socio-cognitive factors related to the COVID-19 crisis build the next larger group of the collected articles (Bernabe-Valero, Blasco-Magraner et al.; Maglić et al.; Podlesek et al.; Tonković et al.). The third group includes papers dealing with preventive and protective behaviors, such as adherence to epidemiological recommendations (Lep et al.; Paiva et al.; Hromatko et al.). A final group of studies conferred deals with the way of mitigating psychological distress (de Rivera et al.; Ozamiz-Etxebarria et al.; Vukičević Marković et al.) during the coronavirus crisis. In the following paragraphs we have grouped these studies according to overarching topic, with their main findings summarized.

## Anxiety and Mental Health

Studies centered around the topic of anxiety and mental health prove that anxiety is found almost universally to be the strongest behavioral feature of life in the COVID crisis. Not surprisingly it sets the main framework for counteracting the negative consequences of the crisis. The following articles show that the key to resilience are supportive and affectionate relationships in different life contexts, gratitude, and empowerment of general coping strategies:

- Fear of COVID-19, Stress, and Anxiety in University Undergraduate Students: A Predictive Model for Depression (Rodríguez-Hidalgo et al.)
- Anxiety and Social Support as Predictors of Student Academic Motivation During the COVID-19 (Camacho et al.)
- The Influence of Personality, Resilience, and Alexithymia on Mental Health During COVID-19 Pandemic (Osimo et al.)
- Mental Health in Affectionate, Antagonistic, and Ambivalent Relationships During the COVID-19 Pandemic: A Latent Profile Analysis (Mehulić and Kamenov)
- The Relationship Between Quarantine Length and Negative Affect During the COVID-19 Epidemic Among the General Population in China: The Roles of Negative Cognition and Protective Factors (Hou et al.)
- The Moderation Effects of Comparative Thinking Between Gratitude and Negative Affect During the COVID-19 Outbreak (Bernabe-Valero, Melero-Fuentes et al.).

## Differential Socio-Cognitive Factors in COVID Crisis

It seems that the consequences of living with anxiety will have a differential effect on different people and it is important to note that a mild subjective cognitive decline may be experienced by some individuals during the epidemic. Individuals characterized by open-minded thinking and general trust in science will be more likely to avoid physical contact, maintain physical hygiene, and support COVID-19 restrictive mitigation policies. These recommendations will also be more appreciated by people who feel more grateful and have a stronger sense of purpose in life, suggesting that these features should be fostered in interventions designed to encourage public coping with the pandemic. The following studies deals with these issues:

- Analytic Thinking and Political Orientation in the Corona Crisis (Maglić et al.)
- Who Believes in COVID-19 Conspiracy Theories in Croatia? Prevalence and Predictors of Conspiracy Beliefs (Tonković et al.)
- The Relationship Between Perceived Stress and Subjective Cognitive Decline During the COVID-19 Epidemic (Podlesek et al.)
- Individual Differences Facing the COVID-19 Pandemic: The Role of Age, Gender, Personality, and Positive Psychology (Bernabe-Valero, Blasco-Magraner et al.).

## Adherance to Recommendations

While individual differences in cognitive and affective responses are important, a coherent and credible communication in all stages of the struggling with pandemic is crucial in forming solidarity with the recommendations and social trust. Still public strategy must keep in mind the individual differences and accommodate the knowledge of these differences in public health strategies when communicating the importance of adherence to recommendations. Psychological mechanisms underlying adherence to pharmacological (vaccination) vs. non-pharmacological measures differ, which should also be considered when planning communication strategy. These are the studies dealing with the above topics:

- One Hundred and Sixty-One Days in the Life of the Homopandemicus in Serbia: The Contribution of Information Credibility and Alertness in Predicting Engagement in Protective Behaviors (Lep et al.)
- Boldness Personality Traits Are Associated With Reduced Risk Perceptions and Adoption of Protective Behaviors During the First COVID-19 Outbreak (Paiva et al.)
- Trust in Science, Perceived Vulnerability to Disease, and Adherence to Pharmacological and Non-pharmacological COVID-19 Recommendations (Hromatko et al.).

## Mitigation of Negative Consequences

Consistent with the universality of anxiety as a key behavioral descriptor of life with a pandemic, studies on the mitigation of pandemic's negative consequences mostly deal with the anxiety mitigation. While some techniques prove to be effective (such as autogenic training and relaxation techniques), it is advised to avoid some self-guided techniques (such as expressive writing) in the context of the COVID 19 pandemic—as suggested by the following studies:

- Autogenic Training Improves the Subjective Perception of Physical and Psychological Health and of Interpersonal Relational Abilities: An Electronic Field Survey During the COVID-19 Crisis in Spain (de Rivera et al.)
- Reduction of COVID-19 Anxiety Levels Through Relaxation Techniques: A Study Carried Out in Northern Spain on a Sample of Young University Students (Ozamiz-Etxebarria et al.)
- Effectiveness of Expressive Writing in the Reduction of Psychological Distress During the COVID-19 Pandemic: A Randomized Controlled Trial (Vukičević Marković et al.).

This Research Collection offers high-quality theoretical insights and empirical findings covering a range of behavioral domains intertwined into our everyday life in the context of pandemic. In a way, the technical details of this issue also speak of life during the pandemic, its immediate effects in terms of our emotional and cognitive experience and behaviors, as well as our ways of coping with its consequences. The pandemic is overall and omnipresent. Fifty-seven authors from 12 countries (Brazil, China, Croatia, Ecuador, Italy, Portugal, UK, United States, Serbia, Slovenia, Spain, Switzerland) and three continents testified about the efforts made world-wide to better understand its impact and mobilize inter- and intra-individual resources in combating its negative consequences.

We have found commonalities of psychological responses of fear, anxiety, conspiracy, resiliency, and support across countries and cultures. We have also seen strong individual and group differences responding to COVID-19 related stress due to age, gender, and culture. This Research Collection provides examples of the many ways in which evolutionary principles can help advance psychological and behavioral science applied in a pandemic context. The Topic provides scientific evidence of effective coping strategies to protect from stress and negative emotions, such as:

- Build trust in science and lower the sense of political helplessness
- Increase optimism, cognition, uncertainty tolerance, and social support
- Mindfulness, relaxation, and writing to reduce stress.

This Research Topic was envisioned as an evidence-based reminder of the times of crisis caused by the new and then little-known coronavirus. By clearly identifying areas of research important for our coping with the pandemic and its effects, this Research Topic has outgrown its primary purpose. It can now be viewed as a tool for generating new valuable research ideas, consolidating the existing findings, and testing future treatment strategies.

## Author Contributions

AV has drafted the article. All authors have contributed to the final revision to the editorial.

## Funding

Part of the effort was supported by United States National Institute of Health AG060608 to YJ.

## Conflict of Interest

The authors declare that the research was conducted in the absence of any commercial or financial relationships that could be construed as a potential conflict of interest.

## Publisher's Note

All claims expressed in this article are solely those of the authors and do not necessarily represent those of their affiliated organizations, or those of the publisher, the editors and the reviewers. Any product that may be evaluated in this article, or claim that may be made by its manufacturer, is not guaranteed or endorsed by the publisher.
